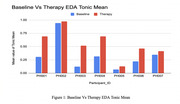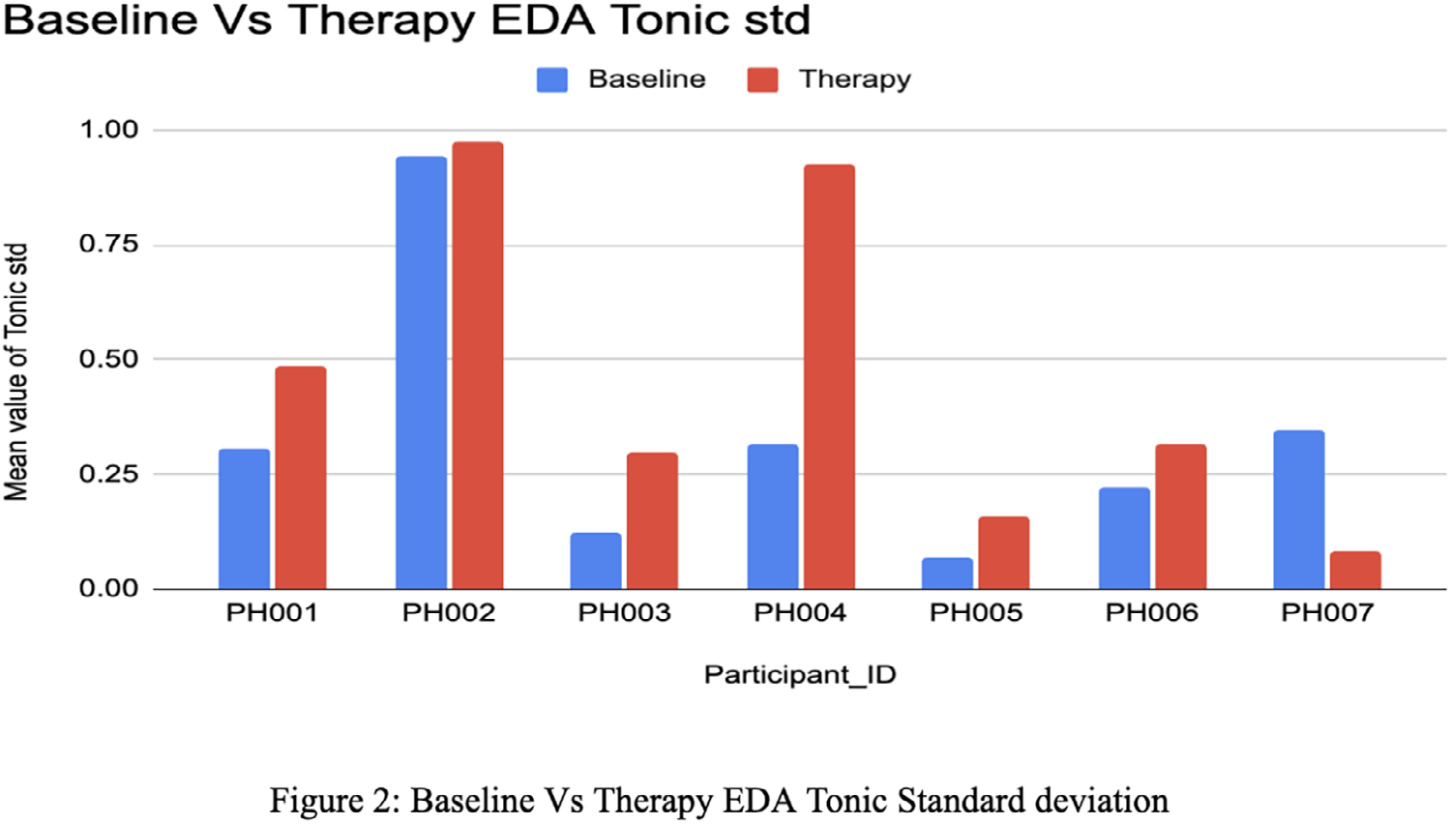# Enhancing Mobility, Cognition, and Engagement in Dementia Care: The Impact of Robotic‐Assisted Physical Therapy

**DOI:** 10.1002/alz70863_110550

**Published:** 2025-12-23

**Authors:** Arshia A Khan, Patricia Khashayar

**Affiliations:** ^1^ University of Minnesota Duluth, Duluth, MN USA; ^2^ University of Minnesota, Minneapolis, MN USA

## Abstract

**Background:**

Dementia affects over 55 million people globally, leading to declines in mobility, cognition, and daily living skills. Structured physical activity can slow cognitive decline by up to 30%, yet traditional therapy engagement is often limited due to apathy. Robotic‐assisted therapy offers an interactive, engaging rehabilitation approach. This study examines its impact on mobility, cognition, and emotional well‐being among memory care residents.

**Methods:**

A seven‐week intervention was conducted with seven memory care residents. A robotic system guided physical therapy exercises to improve mobility, engagement, and well‐being. Electrodermal activity (EDA) data assessed physiological responses, while cognitive and functional changes were measured using the Brief Interview for Mental Status (BIMS) survey before and after the intervention.

**Results:**

**Electrodermal Activity (EDA) Analysis**

**Figure 1: Baseline vs. Therapy EDA Tonic Mean** Baseline EDA was recorded at session start. The tonic component was extracted, and mean values were compared. Therapy sessions led to increased EDA tonic mean values for all participants, indicating greater physiological arousal and engagement.

**Figure 2: Baseline vs. Therapy EDA Tonic Standard Deviation** Tonic standard deviation of EDA was analyzed to assess engagement fluctuations. Therapy sessions resulted in consistently higher EDA standard deviations, suggesting increased responsiveness during robotic‐assisted exercises.

**Conclusion:**

Robotic‐assisted therapy provides a promising, non‐pharmacological approach to improving mobility, cognition, and emotional well‐being in dementia care. Increased EDA values suggest heightened engagement, while BIMS score improvements indicate cognitive benefits. These findings support robotic rehabilitation as a valuable complement to traditional therapy, enhancing quality of life in memory care settings.